# RYBP is important for cardiac progenitor cell development and sarcomere formation

**DOI:** 10.1371/journal.pone.0235922

**Published:** 2020-07-16

**Authors:** Surya Henry, Viktória Szabó, Enikő Sutus, Melinda Katalin Pirity

**Affiliations:** 1 Biological Research Centre, Szeged, Hungary; 2 Doctoral School in Biology, Faculty of Science and Informatics, University of Szeged, Szeged, Hungary; Ann and Robert H Lurie Children's Hospital of Chicago, UNITED STATES

## Abstract

We have previously established that epigenetic regulator RING1 and YY1 binding protein (RYBP) is required for the contractility of embryonic stem (ES) cell derived cardiomyocytes (CMCs), suggesting its essential role in contractility. In order to investigate the underlying molecular events of this phenotype, we compared the transcriptomic profile of the wild type and *Rybp* null mutant ES cells and CMCs differentiated from these cell lines. We identified genes related to ion homeostasis, cell adhesion and sarcomeric organization affected in the *Rybp* null mutant CMCs, by using hierarchical gene clustering and Gene Ontology analysis. We have also demonstrated that the amount of RYBP is drastically reduced in the terminally differentiated wild type CMCs whilst it is broadly expressed in the early phase of differentiation when progenitors form. We also describe that RYBP is important for the proper expression of key cardiac transcription factors including *Mesp1*, *Shh* and *Mef2c*. These findings identify *Rybp* as a gene important for both early cardiac gene transcription and consequent sarcomere formation necessary for contractility. Since impairment of sarcomeric function and contractility plays a central role in reduced cardiac pump function leading to heart failures in human, current results might be relevant to the pathophysiology of cardiomyopathies.

## Introduction

Contractile disorders, such as cardiomyopathy and arrhythmia are often derived from structural malformations of the developing heart and lead to congenital heart defects (CHDs) [1]. Mutations in key cardiac transcription factors such as NK2 Homeobox 5 (*Nkx2-5*), Myocyte Enhancer factor 2C (*Mef2c*) and T-box 5 (*Tbx5*) cause serious problems in heart development and contractile functions [1,2]. Although the major effectors and regulators of cardiac transcription are identified, there are only limited information available about how improper gene expression and structural disorganization result in heart development defects [3–5]. During mammalian heart formation, the early multipotent progenitor cells (MPCs) give rise to the atrial and ventricular cell types, fibroblast cells, endocardial and epicardial cells, cells of the conductive system (sinoatrial, atrioventricular, purkinje fiber cells), the smooth muscle cells of the aorta, artery and the autonomic nerve cells [2]. Several ion channel genes function electro-physiologically in the cardiac conduction system contribute immensely towards the action potential of the contracting heart [6]. As a result of these finely tuned events governed by series of key transcription factors, the developing heart starts beating as early as E8.5–9 in mouse [7]. The underlying molecular events of this complex developmental process can be studied using embryonic stem (ES) cells based *in vitro* differentiation systems. When ES cells are differentiated to cardiac lineages *in vitro*, the first contractile cardiomyocytes (CMCs) appear and start beating when the first functional sarcomeres form, which is happening mostly by the end of the first week.

We have previously reported that mouse ES cells lacking RING1 and YY1 binding protein (RYBP, also known as Death effector domain [DED]-associated factor, DEDAF) could not form beating CMCs *in vitro*. RYBP is an epigenetic regulator and a core member of the non-canonical Polycomb Repressive Complex 1 types (ncPRC1s). Originally ncPRC1s were identified as repressor complexes, but later studies have proven that they can contribute to gene activation as well [8,9]. *In vivo* studies have demonstrated that RYBP is essential for the early mouse embryonic development and the development of organ systems such as the central nervous system, hematopoietic system and the eye [10–12]. By utilizing whole-genome wide transcription analysis we have previously also shown that mouse ES cells lacking RYBP (hereafter mentioned as *Rybp*^*-/-*^ or *Rybp* null mutant) and derivative CMCs express several key cardiac transcription factors (including ISL1 transcription factor (*Isl1*), *Tbx5*) deficiently in comparison to the wild type cells. Moreover, Cardiac troponin T2 (*Tnnt2*), which is a major sarcomeric component of wild type CMCs were amongst the most downregulated genes in the *Rybp* null mutant, suggesting that these gene expression changes were likely to contribute to the contractility defect of the mutant cell line [13].

In this study, we dissected further the molecular events leading to the *in vitro* contractility defect of the *Rybp* null mutant CMCs. By utilising wild type and *Rybp* null mutant mouse ES cells and *in vitro* cardiac differentiation system we compared sarcomere formation and characterised cardiac progenitor formation of the wild type and *Rybp* null mutant CMCs. We applied hierarchical clustering of genome wide transcriptomics to identify genes associated with the impaired contractility of the *Rybp* null mutant CMCs at pluripotent (day 0), early (day 8) and late (day 14) differentiation stages. Our results showed that a large set of genes associated with ion homeostasis, cell adhesion and sarcomere organisation were downregulated in the *Rybp* null mutant CMCs. We investigated the protein abundance of RYBP through the time course of *in vitro* cardiac differentiation and determined whether striated sarcomere and cardiac progenitor pool formation were affected in the *Rybp* null mutant CMCs by using comparative gene expression and protein kinetics studies. Our results show that the RYBP protein is prominently represented at the early phase of cardiac differentiation and that RYBP is nearly absent in the terminally differentiated CMCs in the wild type cultures. We also demonstrate that sarcomeres are not formed properly and several transcription factors important for cardiac progenitor formation are under-represented in the lack of RYBP. These results pinpoint the critical role of RYBP in the early events of cardiac development and consequent sarcomere formation. Our data supports that RYBP is likely required first at early differentiation phases, for the proper cardiac progenitor pool formation.

## Materials and methods

### Cell lines and culture condition

Mouse (129SV/Ola) R1 [14] (hereafter mentioned as *Rybp*^*+/+*^ or wild type) and D11 [10] (*Rybp*^*-/-*^ or *Rybp* null mutant) ES cells were thawed on mitomycin C (Mit C; Sigma, Cat.No M0503) inactivated mouse embryonic fibroblast (MEF) layer and cultured on 0.1% gelatin (Gelatin from bovine skin, Sigma, Cat.No G-9391) coated tissue culture plates [15]. The cells were maintained in ES medium that contained 80% Dulbecco’s Modified Eagle’s medium (DMEM (1x) + GlutaMAX™-I Dulbecco`s Modified Eagle Medium, Gibco, Cat.No 31966–021), 15% (vol/vol) foetal bovine serum (Foetal Bovine Serum, APS, Cat.No S-001A-USDA), 1% glutamine (L-Glutamine 200mM (100x), Gibco, Cat.No 25030–081), 0.1mM non-essential amino acids (MEM Non Essential Amino Acids (100x), Corning, Cat.No 25–025 CIR), 0.1mM 2-mercaptoethanol (2-Mercaptoethanol, Gibco, Cat.No 31350–010), 50 U/ml penicillin/streptomycin (Penicillin/Streptomycin (100x), Gibco, Cat.No 15140–122), 1% sodium pyruvate (Sodium Pyruvate 100mM (100x), Gibco, Cat.No 11360–039) and 1000 U/ml Leukemia Inhibitory Factor (LIF, ESGRO, Chemicon/Millipore, Billerica, MA, USA). ES medium was changed daily. The cells were passaged prior to reaching 70% confluency (approximately every 2 days). ES cells were cultured on 0.1% gelatin coated culture plates for at least three passages before the start of differentiation to deplete potentially present MEF cells from the ES cell culture. Cells were cultured in humidified conditions containing 5% CO_2_ at 37°C.

### *In vitro* cardiac differentiation

For cardiac differentiation, embryoid bodies (EBs) were generated by the hanging-drop (HD) method as previously described [16]. For single-cell suspensions, the cells were dissociated from monolayer culture (day 0) with 0.25% trypsin-EDTA (Trypsin-EDTA (0.5%), Gibco, Cat.No 15400–054). The cells were counted and 1000 cells/ 20 μl differentiation medium (ES medium without LIF) were pipetted on the lid of a bacterial dish (4x10^4^ cells/ml). The dish was filled with Dulbecco’s phosphate-buffered saline (DPBS (1x), Gibco, Cat. No 14190–144) to prevent the droplets from drying out. The cells were allowed to aggregate with the help of gravity by reversing the dish lid. After 2 days, the droplets were collected and individual EBs were plated into a well of a 24-well plate containing 0.1% gelatin-coated coverslips for immunocytochemistry (ICC) experiments and in 0.1% gelatin-coated culture plates for gene expression studies. Differentiation medium was changed every second day. Cardiomyocytes were grown for maximum 21 days and observed for contractility every day under a phase-contrast microscope.

**Fig 1:**
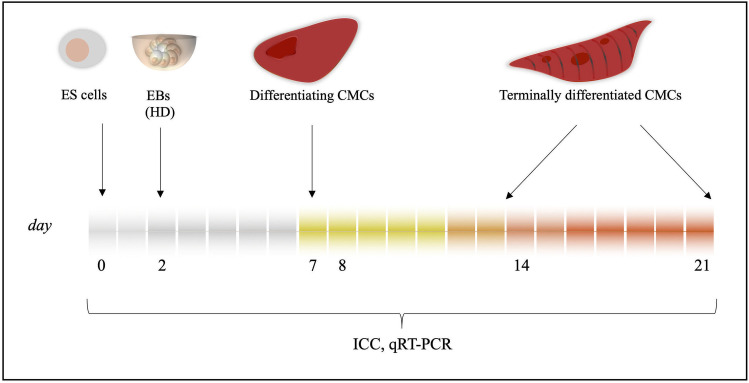
Schematic representation of *in vitro* cardiac differentiation. CMCs were differentiated *in vitro* from ES cells through EB formation by using the HD method. Cardiac colonies were grown for maximum 21 days, sampled for mRNA expression analysis (qRT-PCR) at day 0, 2, 7, 14, 21 and fixed for ICC analysis at day 7, 14 and 21. Samples were derived from day 8 and 14 CMCs for whole genome transcriptomics as described previously [13]. *Abbreviations*: ES cells: Embryonic stem cells, EBs: Embryoid bodies, HD: Hanging drops, CMCs: Cardiomyocytes, ICC: Immunocytochemistry, qRT-PCR: quantitative real-time polymerase chain reaction.

The samples were derived at day 0, 2, 7, 14 and 21 (labelled as d0, d2, d7, d14 and d21, respectively) for further gene expression studies; and at d7, d14 and d21 for ICC (Fig 1), where d0 represents the pluripotent stage, d2 the embryoid body stage, d7 the early and d10, 14 the late cardiac differentiation stages when contractile CMCs are present in abundancy. The expression of *Brachyury* and *Tnnt2* were analysed for cardiac mesoderm and late CMC stages respectively.

### mRNA expression analysis

RNA was isolated from the cell cultures during the time course of *in vitro* differentiation at the designated time points using Gene Jet RNA Purification Kit (Thermo Scientific, Cat.No K0732) according to the manufacturer’s protocol. The RNA was reverse transcribed to cDNA using High Capacity cDNA Reverse Transcription Kit (Applied Biosystems, Cat.No 4368814) as per the manufacturer’s instructions. Quantitative real-time polymerase chain reactions (qRT-PCR) was performed in SYBR® Select Master Mix for CFX (Applied Biosystems, Cat.No 4472942) using Bioer LineGeneK Real-time PCR System machine (Bioer, China). Relative gene expression changes were quantified using the ΔΔCt method. The threshold cycle (Ct) values for each gene was normalized to the expression level of *Hprt*, as internal control. To calculate the fold change, Ct values were compared to undifferentiated samples (d0, *Rybp*^*+/+*^). Experiments were performed in triplicate and repeated from three independent biological samples. See primer sequences in S1 Table.

### Immunocytochemistry

For immunofluorescence staining of the cells, cells were plated onto 0.1% gelatin-coated coverslips and fixed with 4% (v/v) Paraformaldehyde (PFA, Sigma, Cat.No P-6148) for 20 min at RT. Cardiomyocytes were permeabilized with 0.2% Triton X-100 (Sigma, Cat.No T8787) for 20 min at RT and blocked with blocking buffer (5% Bovine Serum Albumin (BSA, VWR Life science, Cat.No 9048-46-8)) for 1h at RT. Cells were washed with DPBS, then incubated with the following primary antibodies at 4°C, overnight. Primary antibodies used for this study include Alpha cardiac muscle actin antibody (GeneTex, Cat.No GTX101876, 1:500), Cardiac troponin T antibody (Abcam, Cat.No ab8295, 1:2000), Cardiac troponin I antibody (DSHB, Cat.No TI-1, 1:200), Tropomyosin antibody (BABRAHAM, Cat.No BT-GB-141, 1:400), sarcomeric Myosin antibody (DSHB, Cat.No MF20, 1:200), Myomesin antibody (DSHB, Cat.No B4, 1:10), Titin antibody (DSHB, Cat.No 9 D10, 1:200), RYBP/DEDAF antibody (Merck-Millipore, Cat.No AB3637, 1:2000). After washing thrice with DPBS the cells were blocked with blocking buffer for 1h, then the cells were labelled with Alexa Fluor 568^®^ Donkey anti-Mouse IgG (H+L) highly cross-adsorbed secondary antibody (Invitrogen, Cat.No A10037, 1:2000 for anti-Troponin T, anti-Troponin I, anti-Myosin-Sarcomere, anti-mMac Myomesin, anti-Titin), Alexa Fluor 488^®^ Donkey anti-Rabbit IgG (H+L) cross-adsorbed secondary antibody (Invitrogen, Cat.No A-21206, 1:2000 for anti-alpha cardiac Actin and DEDAF antibody) and Alexa Fluor 546^®^ Goat anti-Rat IgG (H+L) cross-adsorbed secondary antibody (Invitrogen, Cat.No A-11081, 1:600 for anti-Tropomyosin) for 1h at RT. The cells were washed thrice with DPBS, then rinsed in 4’,6’-diamidino-2-phenylindole (DAPI; Vector Laboratories, Cat.No H-1200) for 20 min. Cells were washed twice with DPBS and mounted in Fluoromount (Fluoromount-G, Invitrogen, Cat.No 00-4958-02). The images were obtained using Olympus LSM confocal microscope (Olympus Corporation). The positive signals were semi-quantified by ImageJ software analysis using RGB measure method.

### Statistical analysis

All experiments were repeated at least three times. Experiments were evaluated by using Student T-test type 3. Means are standard deviation. Values of p < 0.05 were accepted as significant (* p < 0.05; ** p < 0.01; *** p < 0.001).

## Results

### 1. RYBP is abundant in the developing CMCs but nearly absent in the terminally differentiated CMCs

**Fig 2:**
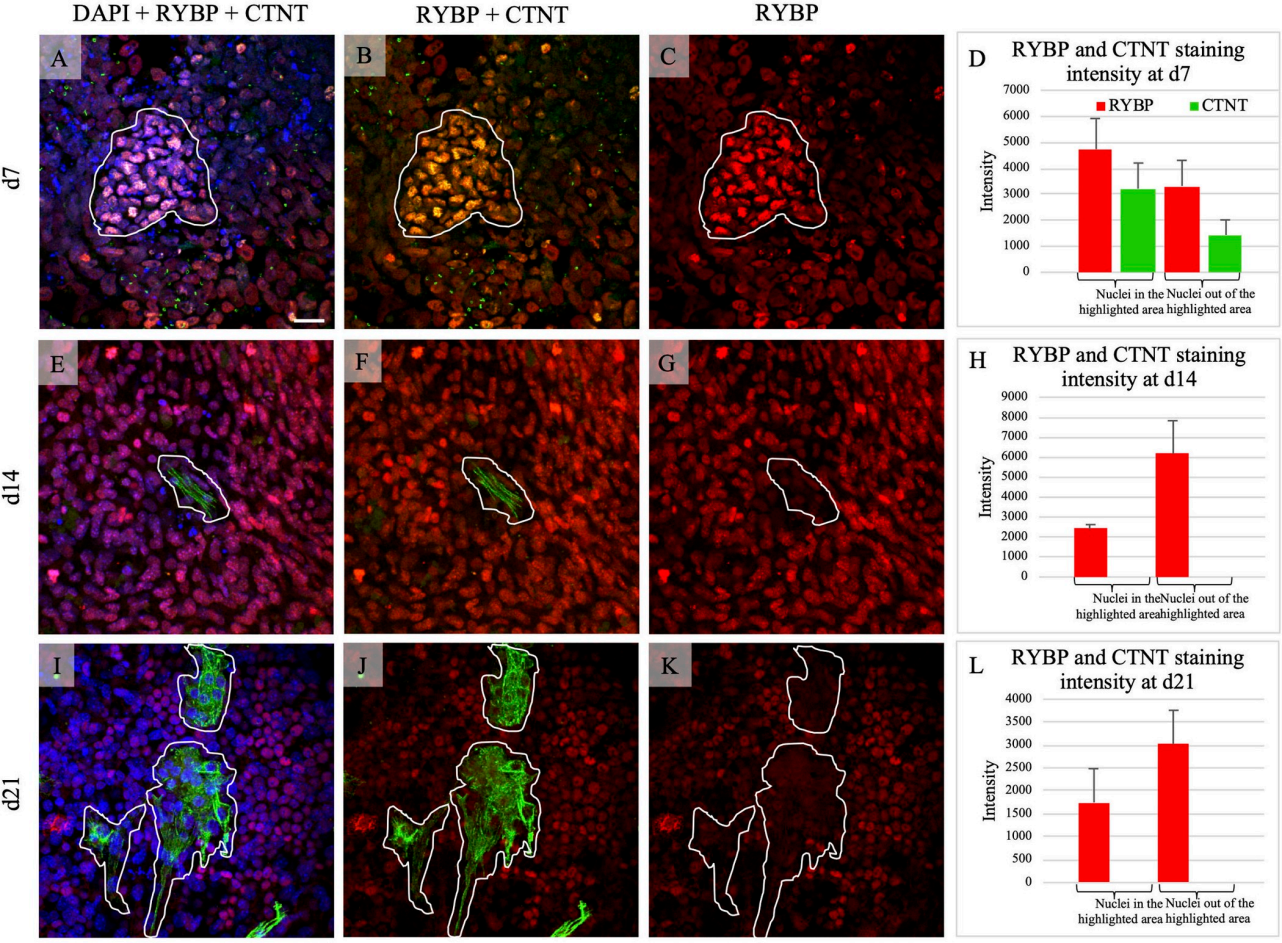
RYBP protein is not present in matured CMCs but expressed at the early stage of *in vitro* cardiac differentiation. Immunocytochemical analysis of RYBP (red) and CTNT (green) protein in wild type (*Rybp*^*+/+*^) CMCs at d7 **(A-D)**, d14 **(E-H)** and d21 **(I-L)**. The areas highlighted in white marks the border of the cells which strongly expressed the CTNT protein. These areas lack striated sarcomeric structures. Semi-quantification of the immunopositive signals where only performed to the nuclei of the cells enclosed in the highlighted region at all time points. Semi-quantification analysis was performed using the RGB measurement tool in ImageJ program **(D, H, L)**. Olympus Confocal IX 81, Obj.: 60 x; Scale bar: 20 μm. Vertical axis indicates the intensity of the fluorescence signal of the immunopositive cells. Means are standard deviation ± SD. *Abbreviations*: DAPI: 4’,6-diamino-2-phenylindol, RYBP: RING1 and YY1 binding protein, CTNT: Cardiac troponin T.

Since contractility depends on the presence of CMCs, first we determined whether RYBP is present in CMCs. We determined the amount and sub-cellular localisation of the RYBP protein in differentiating CMCs during the time course of *in vitro* cardiac differentiation. Wild type ES cells were differentiated for 21 days and fixed in 4% PFA at d7, d14 and d21 where d7 represents the early and d14, d21 represent the terminal stages of cardiac differentiation (see in Materials and methods) (Fig 1). Cardiac cultures were double labelled with anti-RYBP and anti-CTNT antibodies. The anti-CTNT antibody recognizes the product of *Tnnt2*, a classical sarcomere marker of terminally differentiating cardiomyocytes. The sub-cellular localisation of the two corresponding proteins were determined by fluorescent microscopy (see in Materials and methods). As expected, RYBP was mostly found in the nuclei of cells at all stages of *in vitro* cardiac differentiation. At early differentiation stages (d7), RYBP and CTNT co-stained the nuclei of the maturing CMCs (Fig 2A–2C) and the RYBP signal was most pronounced in the CTNT positive cells (Fig 2C, highlighted area). Quantification of the immune-stained early cardiac cultures showed that the RYBP and CTNT signal intensities were nearly two times higher in the nuclei of the cells within the highlighted area than in the surrounding fields (Fig 2D). At d14, CTNT appeared in the striated sarcomeric structures of the cytoplasm (Fig 2E and 2F). Notably, RYBP was nearly absent in the nuclei of CTNT positive cells (Fig 2G). However, there was still detectable RYBP staining in the nuclei of the surrounding CTNT positive CMCs at d14, lacking striated sarcomeres (Fig 2G). Quantification of the fluorescent RYBP and CTNT signals in the nuclei of the cells inside and outside the highlighted areas showed that the RYBP signal was nearly three times lower where CTNT signals marked the striated sarcomeric structures in the cytoplasm of the wild type cultures at d14 (Fig 2H). This pattern was similar and more profound at d21 cultures where RYBP displayed a more modest staining (Fig 2I–2L). These data suggest that RYBP is present in the early phase of differentiation when maturing CMCs are not arranged into striated sarcomeric structures yet. However, RYBP is nearly absent in terminally differentiated cells, which show the matured, striated sarcomeric pattern.

### 2. Transcriptome analysis reveals sarcomeric, ion channel and cell adhesion gene expression changes in the *Rybp* null mutant cells

**Fig 3:**
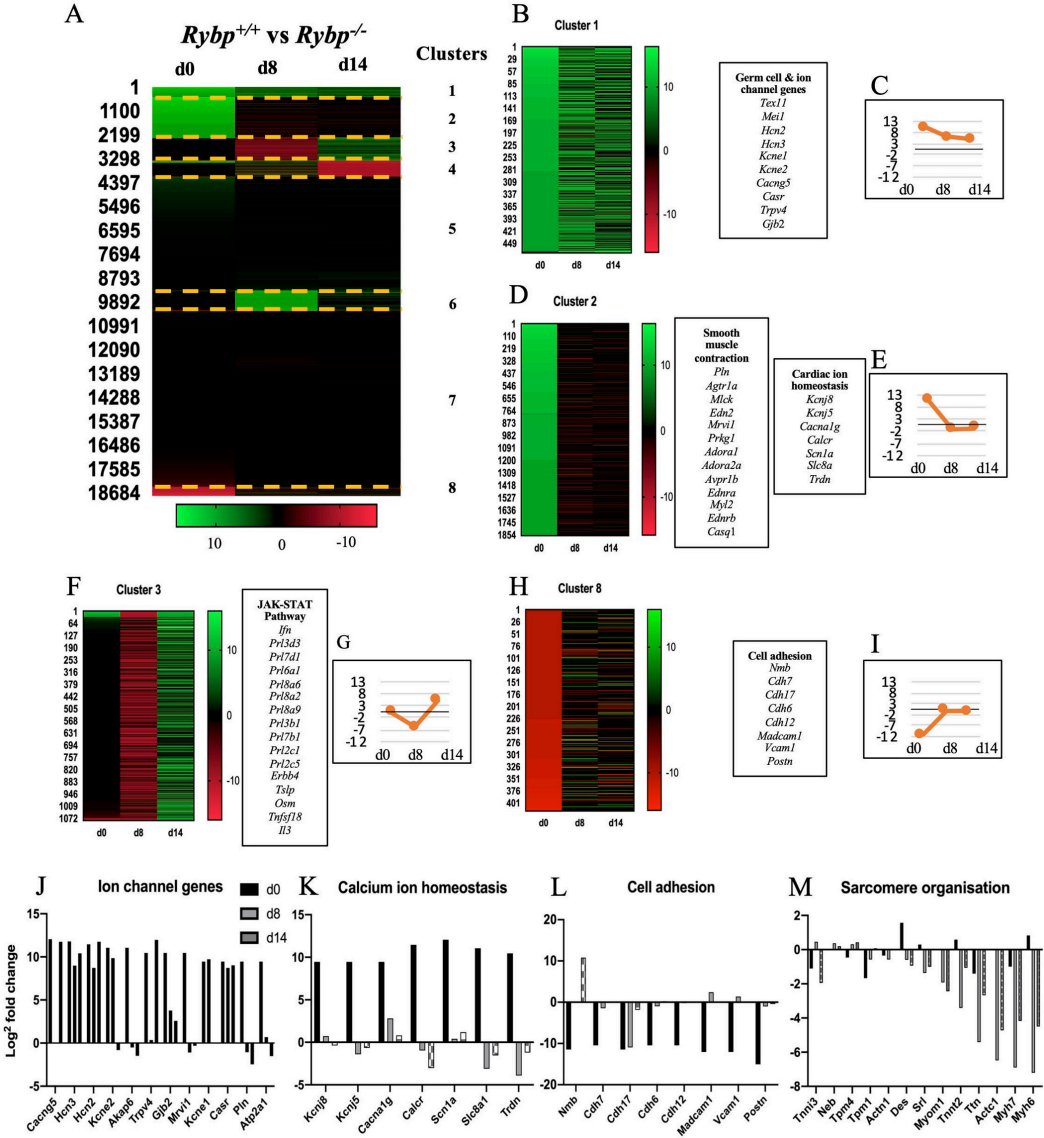
Gene expression changes during *in vitro* cardiac differentiation of the *Rybp null* mutant ES cells and CMCs. **(A)** Heat map of hierarchical clustering of RYBP regulated gene expression changes with significant upregulated (log^2^ fold change ≥2; green colour) and downregulated (log^2^ fold change ≤2; red colour) genes in the *Rybp* null mutant cells. The cluster numbers are listed on the right side of the heat map. **(B and C)** Cluster 1 heat map and tendency graph highlight the upregulated gene set at all three time points i.e. d0, d8 and d14. **(D and E)** Cluster 2 heat map and tendency graph highlight the genes upregulated in d0 only and downregulated in d8 and d14. **(F and G)** Cluster 3 heat map and tendency graph highlight the genes downregulated in d8 and upregulated in d14. **(H and I)** Cluster 8 heat map and tendency graph highlight the genes highly downregulated in d0. The tendency graphs are represented as an average of the overall log^2^ fold change for each time point pertaining to each cluster respectively. **(J)** Bar graph representation of the ion channel genes using log^2^ fold change values from the transcriptome showing constant upregulation of major genes from d0. **(K)** Bar graph representation of the genes involved in maintaining calcium homeostasis show upregulation of major genes in the *Rybp* null mutant ES cells. **(L)** Bar graph representation of genes involved in cell adhesion display constant downregulation of major genes in the *Rybp* null mutant ES cells. **(M)** Bar graph representation of genes involved in sarcomere organization showing downregulation of major genes in d8 and d14 in most cases.

In order to investigate by which mechanism the ablation of *Rybp* results in contractility defects, a detailed comparison of the mRNA transcriptomes across wild type and *Rybp* null mutant mouse ES cells (d0) and derived CMCs (d8, d14) was performed [13]. Samples were collected from the designated time points where d0 represented the pluripotent stem cells stage, d8 the early and d14 the terminal cardiac stages (Fig 1). Hierarchical clustering of the values (log^2^ fold change ≥ 2) between wild type and *Rybp* null mutant ES cells and differentiated CMCs by k-means method using XLSTAT tool revealed 8 distinct gene clusters (Fig 3A). Cluster 1 pertained to genes that were profoundly upregulated (log^2^ fold change ≥ 4) in most stages (i.e. d0, d8 and d14) of *in vitro* cardiac differentiation in the *Rybp* null mutant ES cells (Fig 3B and 3C). These include Hyperpolarization-activated cyclic nucleotide-gated potassium and sodium channel 2 (*Hcn2*) and Hyperpolarization-activated cyclic nucleotide-gated potassium and sodium channel 3 (*Hcn3*) (Figs 3B and S1). High expression of *Hcn2* and *Hcn3* are associated to cause sinoatrial node dysfunction ultimately leading to heart failure [17]. Other cardiac ion channel genes such as Potassium voltage-gated channel Isk-related subfamily member 1 (*Kcne1*), Potassium voltage-gated channel Isk-related subfamily member 2 (*Kcne2*), Calcium channel voltage-dependant gamma subunit 5 (*Cacng5*), Calcium-sensing receptor (*Casr*), Transient receptor potential cation channel subfamily V member 5 (*Trpv5*) and gap junction genes such as Gap junction protein beta 2 (*Gjb2*) were identified to be part of cluster 1 (Fig 3B, 3C and 3J). These genes play essential roles in the maintenance of ion homeostasis in the developing CMCs. In cluster 2 we identified several genes with significantly upregulated expression level in the *Rybp* null mutant ES cells (log^2^ fold change ≥ 4) and decreased expression level at d8 and d14 (log^2^ fold change ≤ 2) (Fig 3D and 3E). Genes that contribute to vascular smooth muscle contraction such as Angiotensin II receptor type 1 (*Agtr1*), Endothelial receptor type A (*Ednra*), Arginine vasopressin receptor 1a (*Avpr1a*), Myosin light chain kinase 3 (*Mylk3* also called as *Mlck*) and Myosin light chain 2 (*Myl2*) that function in vasoconstriction and Adenosine A2a receptor (*Adora2*) that plays role in vasodilation were all part of the same cluster. Genes that are essential in maintaining calcium homeostasis in the developing CMCs such as Potassium inwardly-rectifying channel subfamily J member 5 (*Kcnj5*), Calcium voltage-gated channel T type alpha 1G subunit (*Cacna1g*), Calcitonin receptor (*Calcr*) and Sodium channel voltage-gated type I alpha (*Scn1a*) were also identified in the same cluster showing that key cardiac genes were upregulated from the ES cell stage and these gene expression changes together could potentially lead to the loss of ion equilibrium which is required for the normal formation of CMCs (Fig 3D, 3E and 3K). Cluster 3 contained genes that were extensively downregulated at d8 and upregulated by d14 in the *Rybp* null mutant cells (Fig 3F and 3G). By Gene Ontology (GO) analysis we identified 16 genes that act on the JAK-STAT (Janus Kinase-Signal transducer and activator of transcription proteins) signalling pathway (GO:0046425), that contributes to the normal proliferation and apoptosis of the differentiating cells. In Cluster 4, 5, 6 and 7 we did not identify genes that significantly related to any function connected to cardiac development. In cluster 8, cell adhesion markers such as Cadherin protein 6, 7 and 17 (*Cdh6*, *Cdh7* and *Cdh17*, respectively) and Vascular cell adhesion molecule 1 (*Vcam1*) were identified to be downregulated in the *Rybp* null mutant ES cells (Fig 3H, 3I and 3L). Cell adhesion is a key feature which is required for the proper proliferation and differentiation of various cell types during mammalian heart development.

This analysis also revealed that genes required for sarcomeric organization were remarkably downregulated at d8 and d14 in the mutant cells. Sarcomeric genes such as Myomesin 1 (*Myom1*), Titin (*Ttn*), Actin alpha cardiac muscle 1 (*Actc1*), Myosin heavy peptide 6 cardiac muscle alpha and Myosin heavy peptide 7 cardiac muscle beta (*Myh6* and *Myh7*, respectively) were highly downregulated in the mutant cells (Fig 3M). This analysis shed light on the impairment of sarcomere formation, which can immensely contribute towards the non-contractility phenotype of the *Rybp* null mutant cells as well.

### 3. Lack of RYBP compromises the expression of sarcomeric thin and thick filament coding genes

**Fig 4:**
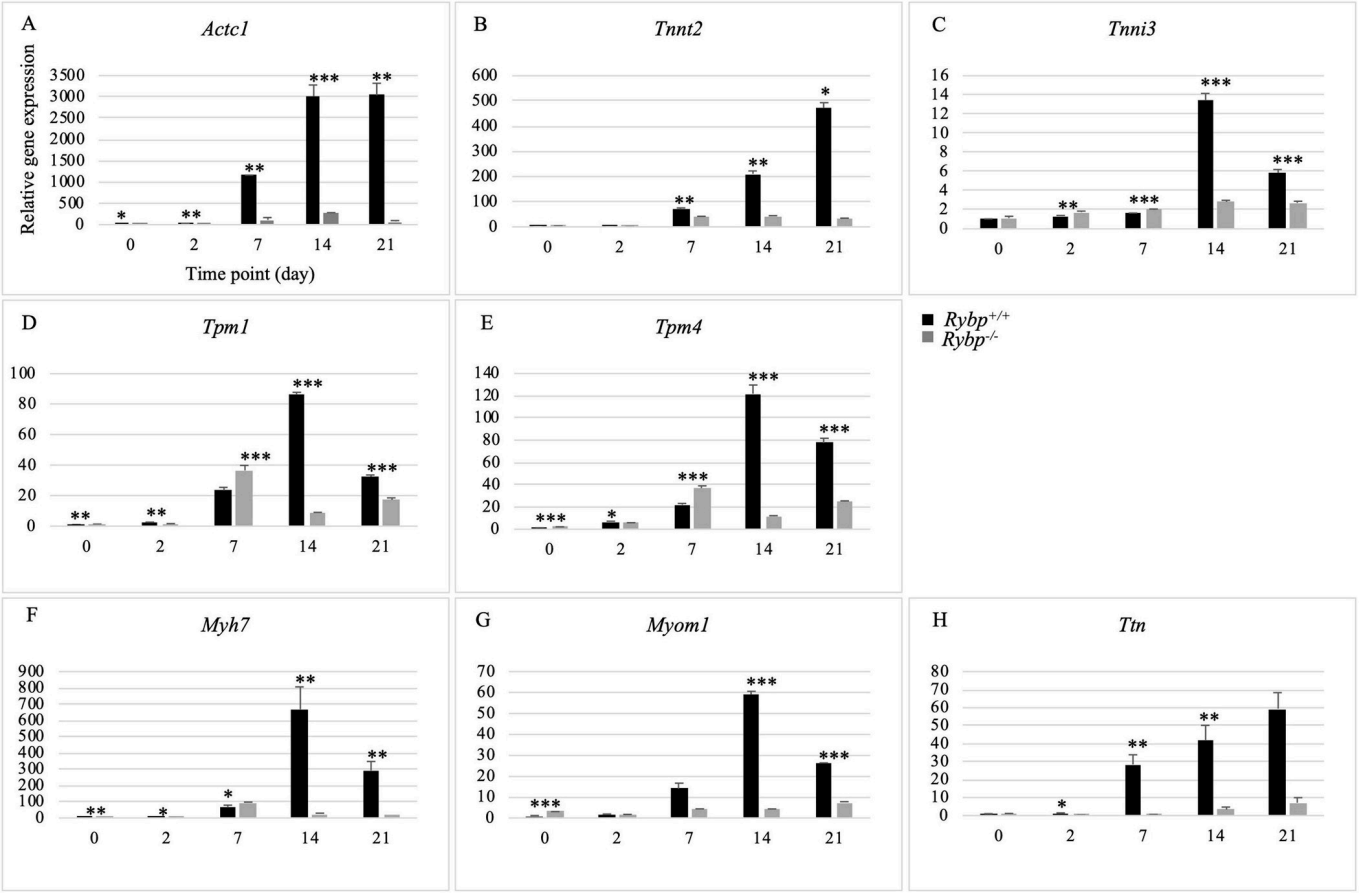
Decreased mRNA expression of thin and thick filament associated genes in the lack of RYBP during *in vitro* cardiac differentiation. Relative gene expression changes of thin-filament associated *Actc1* **(A)**, *Tnnt2* **(B)**, *Tnni3* **(C)**, *Tpm1* **(D)** and *Tpm4* **(E)** and thick-filament associated *Myh7* **(F)**, *Myom1* **(G)** and *Ttn* **(H)** genes were analysed by qRT-PCR. *Abbreviations*: *Actc1*: Actin alpha cardiac muscle 1, *Tnnt2*: Cardiac troponin T2, *Tnni3*: Cardiac troponin I, *Tpm1*: Tropomyosin 1 alpha, *Tpm4*: Tropomyosin 4, *Myh7*: Myosin heavy peptide 7 cardiac muscle beta, *Myom1*: Myomesin 1, *Ttn*: Titin. Means are standard deviation ± SD. Values of p < 0.05 were accepted as significant (* p < 0.05; ** p < 0.01; *** p < 0.001; n = 3). Statistical method: t test type 3.

Since transcriptome analysis indicated a severe downregulation of genes required for sarcomere organization in the *Rybp* null mutant cells, next we compared the relative gene expression changes of the sarcomeric components in the presence and absence of RYBP. Whole cell RNA was extracted from d0, d2, d7, d14 and d21 time points of *in vitro* cardiac differentiation, reverse transcribed and qRT-PCR analysis was performed to examine whether the lack of RYBP affects the expression of sarcomeric components (see in Materials and methods) (Primer list in S1 Table). Gene expression changes were analysed using both wild type and *Rybp* null mutant cells from the designated time points of *in vitro* cardiac differentiation. Our results showed, that *Actc1*, which is the major protein of sarcomeric thin filament, had reduced expression level at all time points of cardiac differentiation in the *Rybp* null mutant cells (Fig 4A). The expression of terminal cardiogenic marker *Tnnt2* was also significantly reduced in the *Rybp* null mutant CMCs compared to the wild type at all examined time points (Fig 4B). Further gene expression analysis revealed downregulation of the other troponin complex members, the Calcium-binding Troponin I cardiac 3 (*Tnni3*) (Fig 4C) in the mutant cells and the expression of the two major cardiac Tropomyosin (*Tpm*) isoforms, Tropomyosin 1 alpha (*Tpm1*) and Tropomyosin 4 (*Tpm4*) were also greatly altered specially at later time points (d14, d21) in the *Rybp* null mutant cells (Fig 4D and 4E).

Similar to the thin-filament components, thick filament marker *Myh7* was expressed to a significantly reduced extent in the mutant cells at all examined time points of cardiac differentiation (Fig 4F). The expression level of the giant sarcomeric component, *Ttn* was found drastically reduced in the mutant cells from d7 and onwards (Fig 4G). Furthermore, *Myom1* expression was about two times lower at d7, nearly ten times lower at d14 and about three times lower at d21 in the *Rybp* null mutant cells (Fig 4H). These results suggested that the gene expression of both the thin and thick filament components were drastically reduced in the absence of RYBP.

### 4. The sarcomeric thin and thick filament proteins are under-represented in the *Rybp* null mutant CMCs

**Fig 5:**
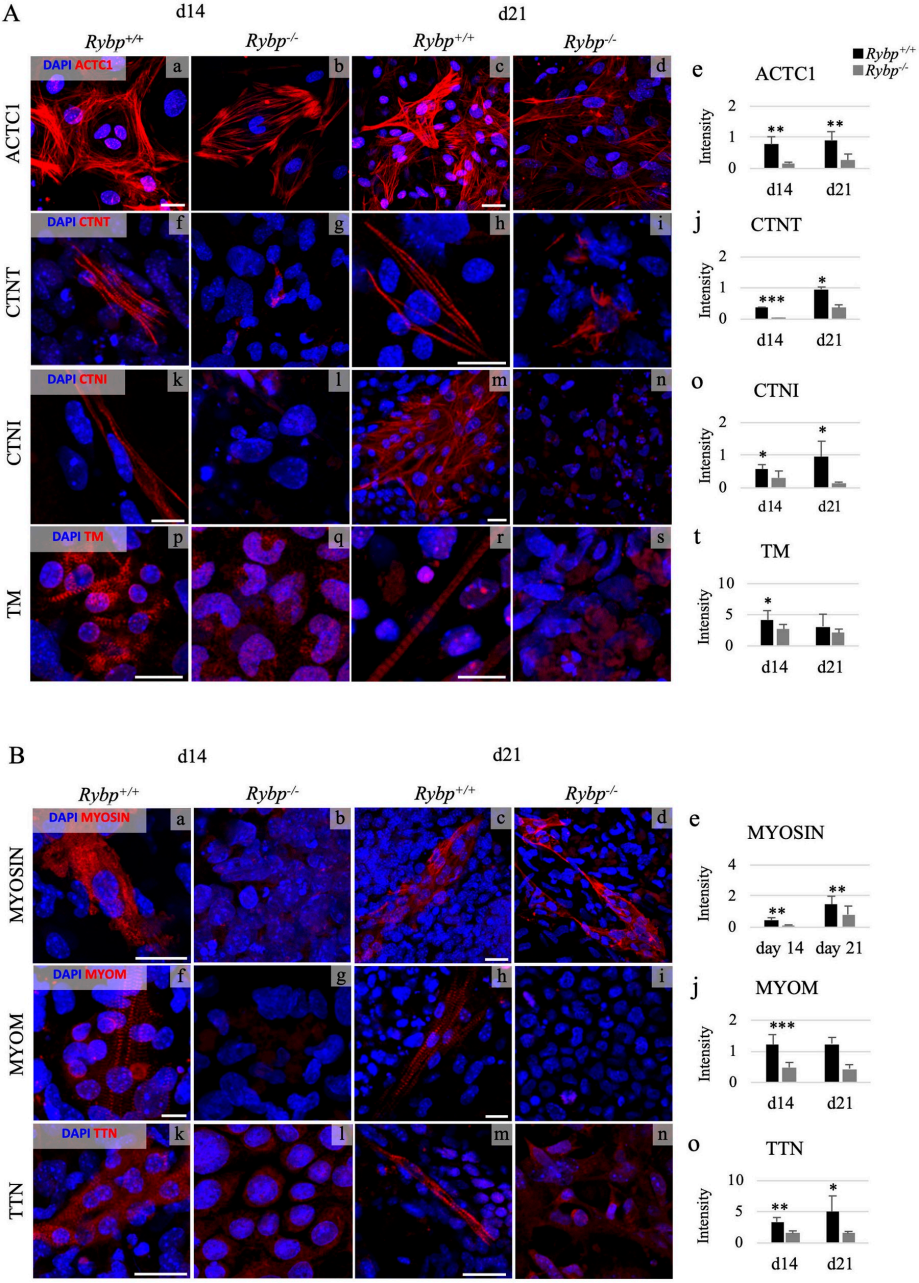
The sarcomeric thin and thick filament associated proteins are disorganized in the lack of RYBP during *in vitro* cardiac differentiation. **(A)** Immunocytochemical analysis of thin filament markers ACTC1**(A a-d)**, CTNT **(A f-i)**, CTNI **(A k-n)** and TM **(A p-s)** in wild type **(A a, c, f, h, k, m, p, r)** and *Rybp* null mutant CMCs **(A b, d, g, i, l, n, q, s)**. Vertical axis indicates the intensity of the fluorescence signal of the immunopositive cells **(A e, j, o, t)**. (**B**) Immunocytochemical analysis of thick filament markers MYOSIN **(B a-d)**, MYOM **(B f-i)** and TTN **(B k-n)** in wild type and *Rybp* null mutant CMCs. Vertical axis indicates the intensity of the fluorescence signal of the immunopositive cells **(B e, j, o)**. Semi-quantification of the signals was performed using the RGB measuring tool in ImageJ program for both the thin and thick-filament proteins. Abbreviations: ACTC1: Actin alpha cardiac muscle 1; CTNT: Cardiac troponin T; CTNI: Cardiac troponin I; TM: Tropomyosin; MYOM: Myomesin; TTN: Titin; d: day; CMC: cardiomyocyte. Olympus Confocal IX 81, Obj.: 60x; Scale bar: Myosin 20 μm, Myom 100 μm/ 50 μm, Ttn 20 μm/ 30 μm. Means are standard deviation ± SD. Values of p < 0.05 were accepted as significant (* p < 0.05; ** p < 0.01; *** p < 0.001). Statistical method: t test type 3.

After revealing the thin and thick filament associated mRNA expression changes, we analysed the subcellular localization and appearance of the corresponding proteins to see if they organised into any striated sarcomeric structures in the two cell lines. Wild type and the *Rybp* null mutant ES cells were differentiated *in vitro* towards CMCs (see Materials and methods). Samples were collected at late timepoints of cardiac differentiation (d14 and d21), when striated sarcomeres are developed, and further processed for ICC (see Materials and methods) (Fig 1). The intensity of the fluorescent signals was measured by ImageJ application and were plotted as graphs (see Materials and methods) (Fig 5A e, j, o, t and Fig 5B e, j, o). Our results showed that the thin filament marker ACTC1 assembled into less dense and filamentous structures in the *Rybp* null mutant cells at both examined time points (Fig 5A a–d). This result corresponded to the reduced gene expression level (Fig 4A). Semi-quantitative analyses of the individual immune-positive cells confirmed up to four times decrease of the ACTC1 protein level in the *Rybp* null mutant cells when compared to the wild type (Fig 5A e). Next, we analysed the two troponin complex members, CTNT and CTNI (protein translated from *Tnni3*). We identified that neither CTNT (Fig 5A f–i) or CTNI (Fig 5A k–n) appeared as part of any organized sarcomere in the *Rybp* null mutant CMCs, the proteins were first seen at d14 distributed in a diffused subcellular localisation in the cytoplasm (Fig 5A f and g) and later on at d21 a disorganized shorter filament staining was observed (Fig 5A h and i). This result also correlates with the reduced mRNA levels of *Tnnt2* and *Tnni3* (Fig 4B and 4C). Semi-quantification of CTNT protein level shows that the staining intensity was eight times lower at d14 and two times lower at d21 and in case of CTNI the staining intensity showed two times lower and five times lower protein levels at d14 and d21 in the *Rybp* null mutant cells respectively (Fig 5A j and o). Furthermore, TM (translated from *Tpm*) did not show as part of any organized sarcomeric structure in the *Rybp* null mutant CMCs neither at d14 (Fig 5A p and q), nor at d21 (Fig 5A r and s), whilst the wild type cells exhibited the expected striated pattern (Fig 5A p and r). The TM protein level was two times lower in the *Rybp* null mutant CMCs (Fig 5A t). These results correlate to the reduced mRNA levels at d14 and d21 (Fig 4D and 4E). In summary, these results suggested that thin filament component protein production was strongly impaired by the absence of functional RYBP.

Next, we compared the amount and subcellular distribution of thick filament associated proteins in the wild type and *Rybp* null mutant cells. We identified normal, striated Myosin structure in the wild type cultures at d14, while there was just a limited amount of protein present in the *Rybp* null mutant cells (Fig 5B a and b). Myosin signals were seen as dense, filamentous and striated pattern in the wild type CMCs at d21, but in the *Rybp* null CMCs it was less filamentous and lacked any striated structure (Fig 5B c and d). These data correlated with the reduced gene expression level of *Myh7* mRNA in the *Rybp* null mutant cells (Fig 4F). The staining intensity of individual immunopositive cells for Myosin protein clearly displayed the decreased protein levels (Fig 5B e). The subcellular organization of Myomesin (MYOM) appeared as displaying sarcomeric structure in the wild type cultures but did not exhibit any organized sarcomere structures in the *Rybp* null mutant CMCs at neither d14 nor d21 (Fig 5B f–i). Moreover, there were hardly any detectable positive MYOM signal in the *Rybp* null mutant cells (Fig 5B j). The staining intensity of individual immunopositive cells correlated to the reduced mRNA level of *Myom1* gene (Fig 4B). The immunostaining of TTN showed that there were well-organized sarcomeric structures in the wild type cells, whilst the *Rybp* null mutant CMCs did not have any structured sarcomeres in both time points and the protein showed diffuse dispersion in the cytoplasm (Fig 5B k–n). Measuring the intensity of signals confirmed that TTN was present only in reduced amount in the *Rybp* null mutant CMCs (Fig 5B o) and this observation is consistent with the reduced mRNA expression level of *Ttn* gene (Fig 4C).

These results suggested that the thin filament and thick filament components of the sarcomere were affected in the absence of RYBP.

### 5. Cardiac progenitor formation is immensely affected in the *Rybp* null mutant cells

**Fig 6:**
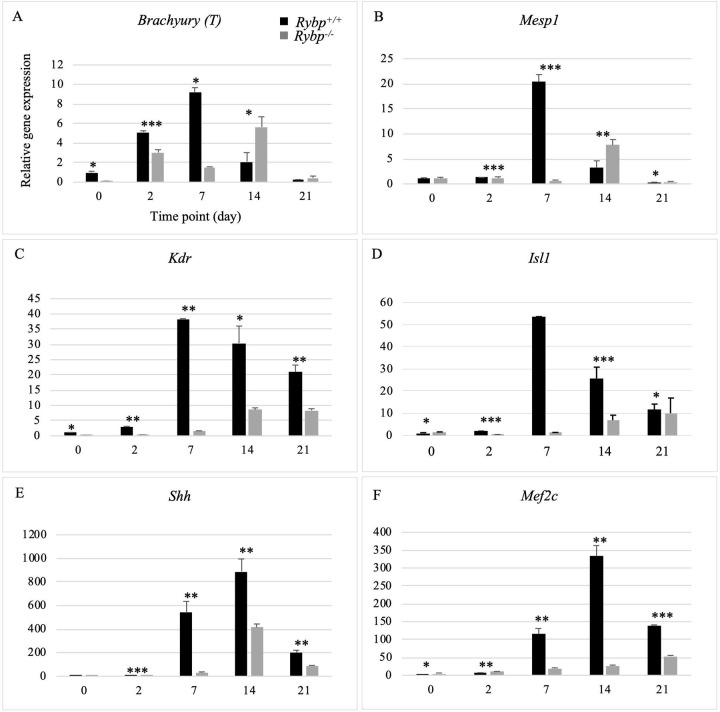
Cardiac progenitor formation is impaired in the *Rybp* null mutant cells. Relative gene expression changes of cardiac progenitor markers *Brachyury* **(A)**, *Mesp1* **(B)**, *Kdr* **(C)**, *Isl1* **(D)**, *Shh* **(E)** and *Mef2c* **(F)** revealed deficiency in the cardiac pool formation in the *Rybp* null mutant cells. *Abbreviations*: *Mesp1*: Mesoderm posterior 1, *Kdr*: Kinase insert domain protein receptor, *Isl1*: ISL1 transcription factor, *Shh*: Sonic hedgehog and *Mef2c*: Myocyte enhancement factor 2c. Means are standard deviation ± SD. Values of p < 0.05 were accepted as significant (* p < 0.05; ** p < 0.01; *** p < 0.001; n = 3). Statistical method: t test type 3.

Since cardiac lineage specific gene expressions were affected from as early as ES cell stage, we wondered if the cardiac progenitor formation was affected in the *Rybp* null mutant cells. Proper cardiac progenitor formation contributes extensively towards differentiation of CMCs. Gene expression changes were checked by qRT-PCR using both wild type and *Rybp* null mutant cells during the time course of *in vitro* cardiac differentiation for the expression of cardiogenic mesodermal markers *Brachyury* (*T*) and Mesoderm posterior 1 (*Mesp1*) and early cardiac markers Kinase insert domain protein receptor (*Kdr* also called as *Flk-1*), *Isl1*, Sonic hedgehog (*Shh*) and *Mef2c* which mark the formation of the first and second heart field (FHF & SHF) *in vivo* (Primer list in S1 Table). In the wild type cultures the expression of *T* and *Mesp1* mRNA was increasing gradually at the beginning of differention, peaked by d7 and declined during the terminal stages (d14 and d21). In the *Rybp* null mutant cells the expression of both *T* and *Mesp1* were deficient showing the highest difference at d7 when compared to the wild type. At d14, the expression of *T* and *Mesp1* displayed a delayed kinetic shift in the *Rybp* null mutant cells (Fig 6A and 6B). The expression of *Kdr* and *Isl1* was pronounced at d7, then declined and gradually went down by d21 in the wild type cells. In the *Rybp* null mutant cells the expression levels never reached the levels of the wild type cells (Fig 6C and 6D). Cardiac progenitor markers *Shh* and *Mef2c* displayed similar expression kinetics with profound expression seen from d7. In d14 their expression was the highest and their expression levels went down by d21 in the wild type cells. In the *Rybp* null mutant cells *Shh* and *Mef2c* were deficiently induced during all time points of *in vitro* cardiac differentiation (Fig 6E and 6F).

Existing ChIP-seq tracks [18] of RYBP and RNF2 display binding peaks at the *Mesp1*, *Shh*, *Nkx2-5* and *Isl1* genomic locus (S2A–S2D Fig and S3 Table) and the binding peaks of RYBP and RING finger protein 2 (RNF2, also known as RING1B) were occupied at the CpG islands of *Mesp1*, *Shh*, *Nkx2-5* and *Isl1* genomic locus. Integrative heatmap of RYBP and RNF2 ChIP-seq binding peaks and the global CpG islands exhibit global occupancy of RYBP and RNF2 at the CpG islands in mesoderm precursor cells. RYBP seemed to show more condense binding at the CpG islands than RNF2 (S2E and S2F Fig and S4 Table). These results suggest that in the lack of RYBP, cardiac progenitor formation is impaired, early cardiac progenitor markers are inappropriately downregulated suggesting that the insufficient progenitor pool can limit consequent CMC development and contractility.

## Discussion

Contractility depends on functional sarcomeres, which develop via finely tuned mechanisms of multiple transcription factors and events including signalling pathways and structural formations. To unreveal the undergoing molecular events of contractility disorders is not only crucial for understanding the mechanisms of development, but it is important for early diagnosis of heart diseases and their treatment. In the current study we provide evidence about the importance of epigenetic regulator RYBP in CMC development including sarcomere and early cardiac progenitor formation. We provide evidence for the first time that CMC development is impaired and functional sarcomeres cannot be formed in the lack of RYBP suggesting its role in the development of cardiac contractility and related disease conditions. Epigenetic regulators have key role in organogenesis, but their function in cardiac development is still elusive. Histone deacetylases regulate contractility of the heart in mice [19] and members of the Polycomb Repressive Complex 2 (PRC2) are required for proper spatiotemporal regulation of cardiac gene expression and cell growth [20]. Members of the ncPRC1s, regulate mesodermal precursor fate and lineage specification [18] but their role in cardiac progenitor formation, lineage commitment and contractility are not defined yet.

Elucidating the role of RYBP in cardiac differentiation is important as RYBP is abundantly present in the embryonic mouse heart *in vivo* [13]. In the current study, we showed that RYBP is the most abundant at the early developmental stage CMCs *in vitro* (d7) where CTNT is also present (Fig 2A–2C), and that RYBP is nearly absent in the terminal stages of cardiac differentiation where the cells exhibited the striated pattern of CTNT (Fig 2E and 2I) suggesting that the role of RYBP is more prominent during the early stages of cardiac differentiation. CTNT is one of the major structural components of CMCs and is expected to appear as a cytoplasmic component. The nuclear staining of CTNT in d7 differentiating wild type CMCs is unexpected and is still not fully understood [21]. CTNI, which is another member of the cardiac troponin complex was shown to be present in the nucleus in primary cultures and it relocalized to the cytoplasm during consequent myogenic differentiation [22]. Since the cardiac troponin complex comprising of CTNI and CTNT along with Cardiac troponin C1 (CTNC) function together in modulating actin-myosin movement, it is possible that CTNT can also be transported from the nucleus to the cytoplasm during the process of CMC maturation.

Hence, the absence of RYBP is already manifested at the stage of progenitor formation it is not unexpected that the effect alters late events like sarcomeric structure formation as well. While RYBP is a well-known member of the ncPRC1s, its functions towards cardiac ion channel genes such as *Hcn2*, *Hcn3*, *Kcne1*, *Kcne2* and *Trpv5* seemed classical in the ES cell stage as they were over ten times upregulated in the *Rybp* null mutant ES cells. Upregulation of these genes alone are already shown to cause contractility defects [17,23]. *Cacna1g*, *Agtr1*, *Ednra*, *Avpr1* and *Adora2* are other genes highly upregulated in the *Rybp* null mutant ES cells (Fig 3D) suggesting that RYBP might be amongst the factors required for the repression of these lineage specific genes. The upregulation of these ion channel genes could lead to hyperpolarization in the *Rybp* null mutant ES cells and can cause breakdown in the differentiation process eventually contributing to impairment in contractility [24].

We also demonstrated that RYBP is required for sarcomere development. RYBP has not been previously described to be required for the proper assembly of sarcomeric components. During cardiac colony formations, the cytoplasm of the mutant cells showed diffused distribution of sarcomeric components at d14 and d21 of *in vitro* cardiac differentiation, while the wild type cells had striated sarcomeric organization. Gene expression analysis of undifferentiated ES cells, as well as early (d7) and matured (d14) CMCs demonstrated the downregulation of both thin filament (*Actc1*, *Tnnt2*, *Tnni3*, *Tpm1*, *Tpm4*) and thick filament markers (*Myh7*, *Myom1*, *Ttn*) and associated structural proteins that are critical for the proper sarcomere formation (Fig 4A–4H). The immunocytochemical analysis of these proteins further confirmed the thin and thick filament components were disorganized and irregularly formed in the *Rybp* null mutant CMCs (Fig 5A and 5B). This suggests that RYBP is essential for the formation of the major components i.e. thin and thick filaments of the sarcomeres. Deficiency in gene expression and assembly of key sarcomeric components can individually cause impairment in cardiac differentiation. Deletion of thin filament markers *Tnnt2* and *Tnni3* and thick filament marker *Ttn* in mice caused severe cardiomyopathy pinpointing the relevance that RYBP may function in the development of cardiomyopathy in humans as well [25–27].

**Fig 7:**
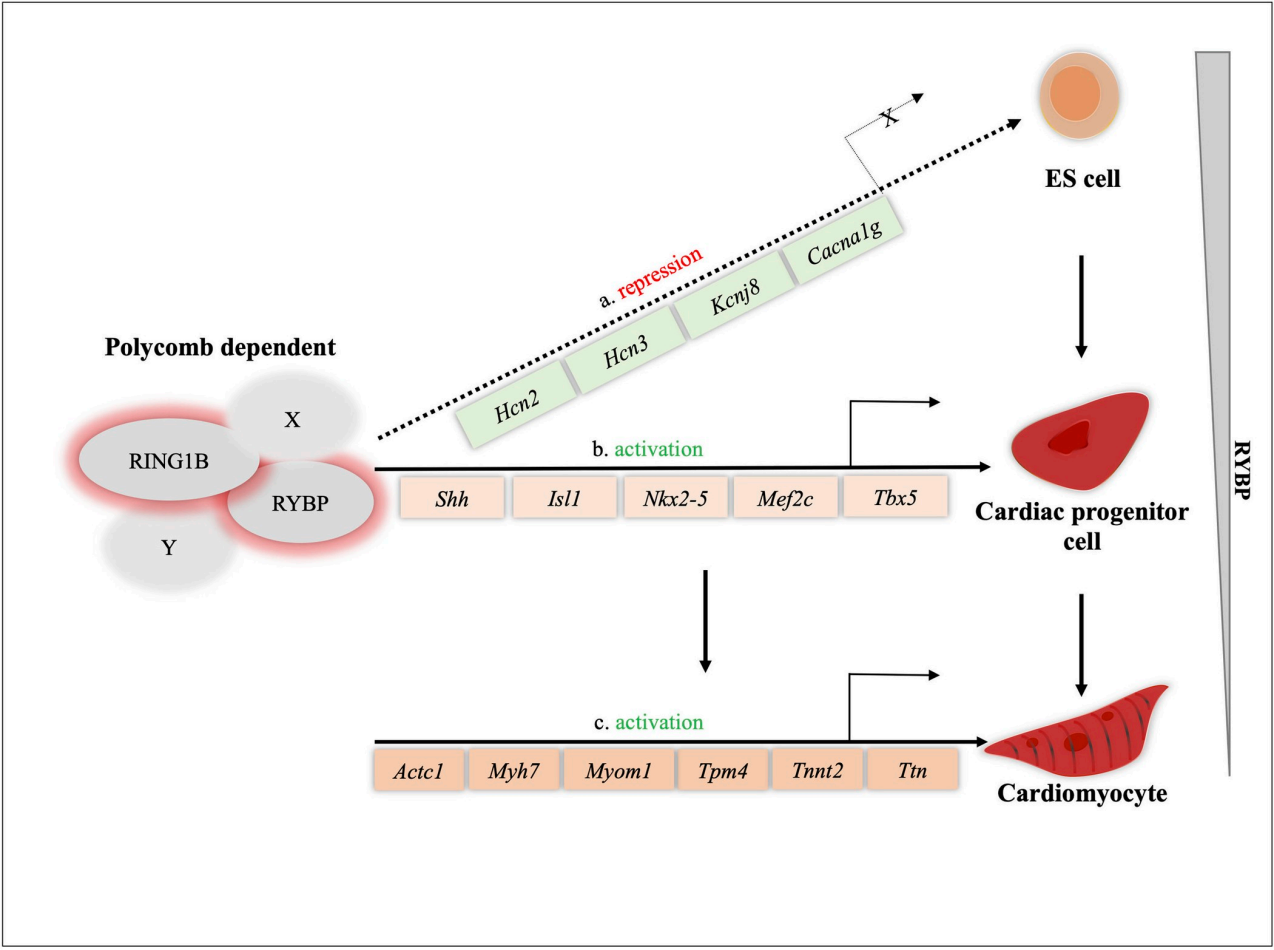
Hypothesized role of RYBP during *in vitro* cardiomyogenesis. Schematic representation showing possible regulatory functions of RYBP during *in vitro* cardiac differentiation. RYBP can exert both repression **(a)** and activation **(b, c)** functions during cardiac morphogenesis. RYBP as the member of ncPRC1s along with RING1 can repress key lineage genes such as ion channel genes during the ES cell stage **(a)**. As a repressor RYBP can repress ion channel genes such as *Hcn2*, *Hcn3*, *Kcnj8 and Cacna1g*. As an activator, RYBP can activate key cardiac transcription factors, like *Shh*, *Isl1*, *Nkx2-5*, *Mef2c* and *Tbx5* and that are required for normal cardiac progenitor formation **(b)**. RYBP and RING1 can target together *Tpm4* and *Myh7*, and as they were described as essential components for proper cardiac morphogenesis **(c)**, whilst other sarcomeric genes such as *Actc1*, *Myom1*, *Tnnt2* and *Ttn* could be regulated by NKX2-5, MEF2C and TBX5.

By examining the mRNA levels of cardiac progenitor markers such as *T*, *Mesp1*, *Kdr*, *Isl1*, *Shh and Mef2c* we suggest that the cardiac progenitor formation is also greatly affected in the *Rybp* null mutant cells. In the lack of RYBP, all the examined progenitor markers exhibited a huge difference in their expression levels at d7, the stage which is critical for cardiac progenitor formation in the wild type cells. The normal expression of cardiac progenitor transcription factors such as *Mesp1*, *Shh*, *Isl1*, *Shh* and *Mef2c* are shown to play essential roles for the formation of the structural and functional units of contractile CMCs [28–32]. They also collectively function in the formation of the first and second heart fields *in vivo* [33]. These genes are also shown to be enhanced in the pulsating primitive heart tube suggesting the downregulation of these genes are crucial in triggering the catastrophic changes that cause non-contractility from as early as d7 in the *Rybp* null mutant cells [28–32]. Previous studies have determined that during cardiac precursor formation RYBP can target genes crucial for cardiac progenitor formation. Furthermore, these genes are direct targets of both RYBP and RNF2 as well [18] (S2 Table and S2A–S2D Fig) (Fig 7A). The binding peaks of both RYBP and RNF2 at *Mesp1*, *Shh*, *Nkx2-5* and *Isl1* genomic locus displayed their co-occupancy at the CpG Islands (S2A–S2E Fig). The downregulation of *Mesp1*, *Shh*, *Nkx2-5* and *Isl1* in the *Rybp null* mutant cells and the direct binding of RYBP at their genomic region present a seemingly plausible ncPRC1 mediated regulation of these genes (S4 Table). These data suggest that RYBP is important for the proper induction of differentiation at progenitor stage when precardiac cells mature towards cardiomyocytes by inducing the expression of cardiac-specific genes.

Whilst RYBP and RNF2 can directly target sarcomeric genes *Myh7*, *Tpm1* and *Tpm4*, it is more likely that the majority of genes required for sarcomere organization are activated by the cardiac transcription factors that include NKX2-5, GATA Binding Protein 4 (GATA4), MEF2C and TBX5 [34,35]. Co-occupancy of NKX2-5, GATA4, MEF2C and TBX5 are identified to bind to the promoters of key cardiac genes essential for the formation of CMCs including the sarcomeric components [34,36] (S3 Table) (S2G Fig) (Fig 7B). Therefore, the lack of RYBP may also represent an indirect effect on the formation of sarcomeres by regulating cardiac transcription factors.

The precise mechanism by which RYBP exerts its functions throughout development is still not thoroughly known. The activity of RYBP containing ncPRC1 complexes is strongly influenced by their subunit composition and stoichiometry [9,37]. ncPRC1s contain RING finger protein 1 (RING1 also known as RING1A), RNF2, Polycomb Group Ring Fingers (PCGFs) and RYBP or YY1 associated factor 2 (YAF2) as the core subunits instead of Chromobox (CBX) and Histone phosphorylation (HPH) subunits of PRC1s. Notably, ncPRC1 subunits can associate with a versatile set of accessory proteins, which often specifies the developmental function of the complex. RYBP can bind YY1, a repressor of sarcomeric gene expression [38]. This way ncPRC1s recruiting YY1 has the potential to regulate sarcomeric genes as well. Knockdown of ncPRC1.2 complex member Polycomb group ring finger 2 (PCGF2 also known as MEL18) is shown to regulate cardiomyogenesis. By utilising RNA-sequencing and ChIP-sequencing (ChIP-seq) approach PCGF2 is shown to exert repressor functions in the ES cell stage and later revealed to exert both activation and repressor functions [18]. Most of the upregulated ion channel gene set in the *Rybp* null mutant ES cells identified from the transcriptomic analysis (Fig 3B–3E) were also direct targets of RYBP and RNF2 as seen in the previously described ChIP-seq analysis [18] whereas certain genes such as *Hcn2*, *Kcnj8*, *Cacna1g* and *Calcr* were targets of PCGF2 as well suggesting that these genes could be regulated in a polycomb dependent manner in the ES cell stage. Although there is emphasis on the role of PCGF2 in cardiomyogenesis, no efforts were taken to investigate the effect of the lack of PCGF2 in sarcomere organization [18]. The question whether RYBP mediates its functions via ncPRC1 during CMCs differentiation still remains opened. The fact that the absence of RYBP caused impaired cardiac progenitor formation suggests that RYBP likely acts as an activator in the process. It is possible that RYBP could either act via polycomb dependent or polycomb independent manner, regulating key lineage determinant factors which are critical for progenitor formation in wild type conditions. Moreover, RYBP can perform more than one function being a moonlighting protein, and member of several multiprotein complexes besides ncPRC1s [9,37]. This brings up the possibility that RYBP can also function as a part of other multimeric protein complexes other than ncPRC1s and regulate cardiac progenitor as well as sarcomere formation consequently (Fig 7).

In order to establish the role of RYBP during heart development and disease formation more emphasis will be required to be put on *in vivo* animal models or three-dimensional tissue culture studies. Due to the fact that the phenotype of the *Rybp* null mutant mice are early embryonic lethal, the conventional knockout mouse models are not suitable for answering the role of *Rybp* in mammalian heart development. More emphasis will also be required to be put on conditional mouse models to clarify the exact role of RYBP in heart development.

Taken together we provide evidence for the first time that RYBP is important for CMC development especially at the early phase when cardiac progenitors and sarcomeric filaments form.

## Supporting information

S1 Fig Lineage specific ion channel genes are upregulated in the early time points of cardiac differentiation. (TIF)

S2 Fig Co-occupancy of RYBP and RNF2 at the CpG islands of key cardiac genes and the co-occupancy of NKX2-5, GATA4, MEF2C and TBX5 at the *Tnnt2* regulatory regions as shown by indicating previously published ChIP-seq peaks. (TIF)

S1 Table qRT-PCR primers. (XLSX)

S2 Table List of genes in each cluster connecting to Fig 3A. (XLSX)

S3 Table ChIP-seq binding targets of RYBP, RING1B, NKX2-5, GATA4, MEF2C, ISL1 and TBX5 amongst key cardiac genes suggest a polycomb mediated regulation of *Shh*, *Nkx2-5*, *Gata4*, *Mef2c*, *Isl1 and Tbx5* by RYBP. (XLSX)

S4 Table List of genes deregulated in the *Rybp*^*-/-*^ null mutant at d8 of RYBP in MES cells from GSM1657391. (XLSX)
