# Supplementary figures and images for: RYBP is important for cardiac progenitor cell development and sarcomere formation
Source: PLoS One. 2020 Jul 16;15(7):e0235922. doi: 10.1371/journal.pone.0235922 (PMC7365410; doi:10.1371/journal.pone.0235922)

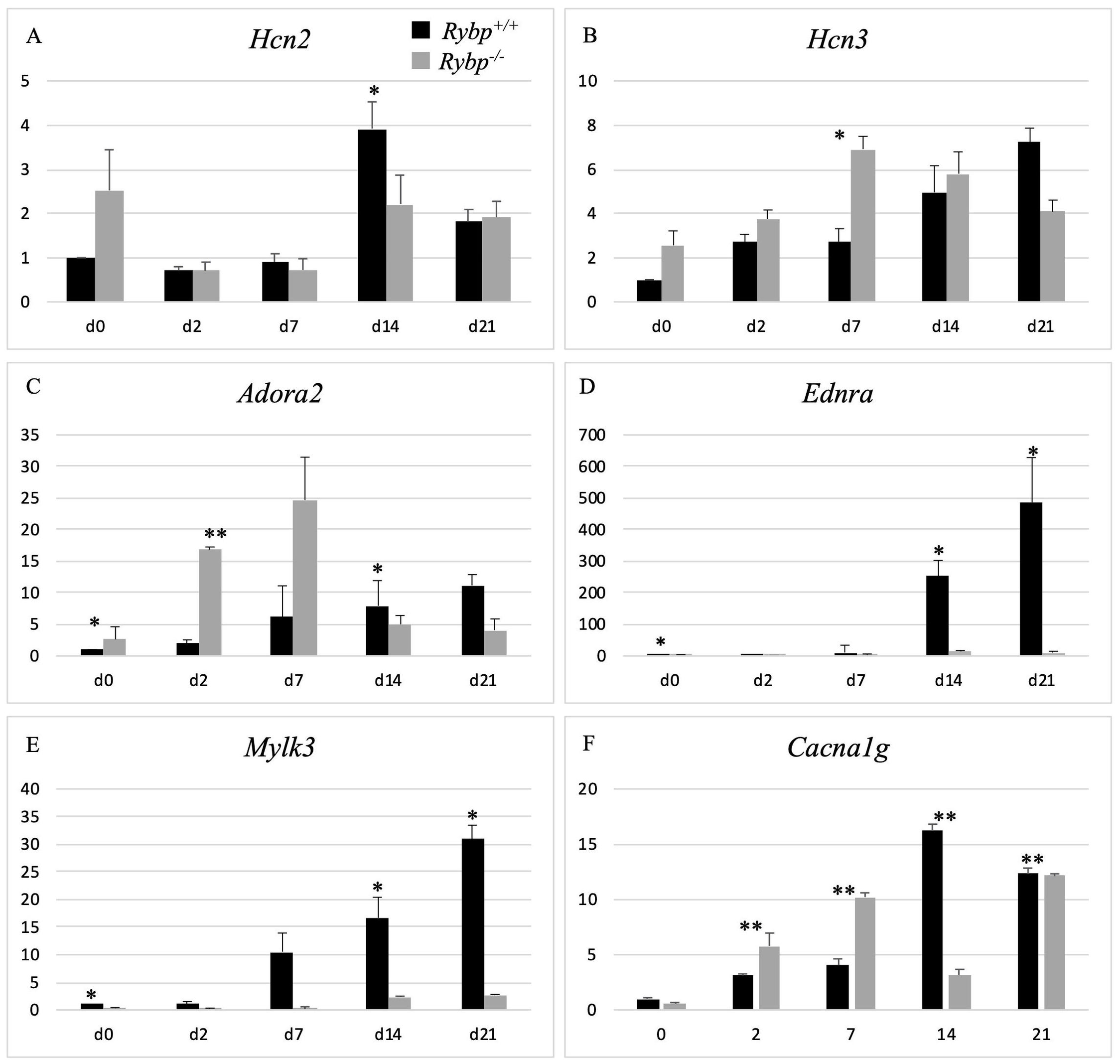

Supplement: S1 Fig — (TIF) [file pone.0235922.s001.tif]

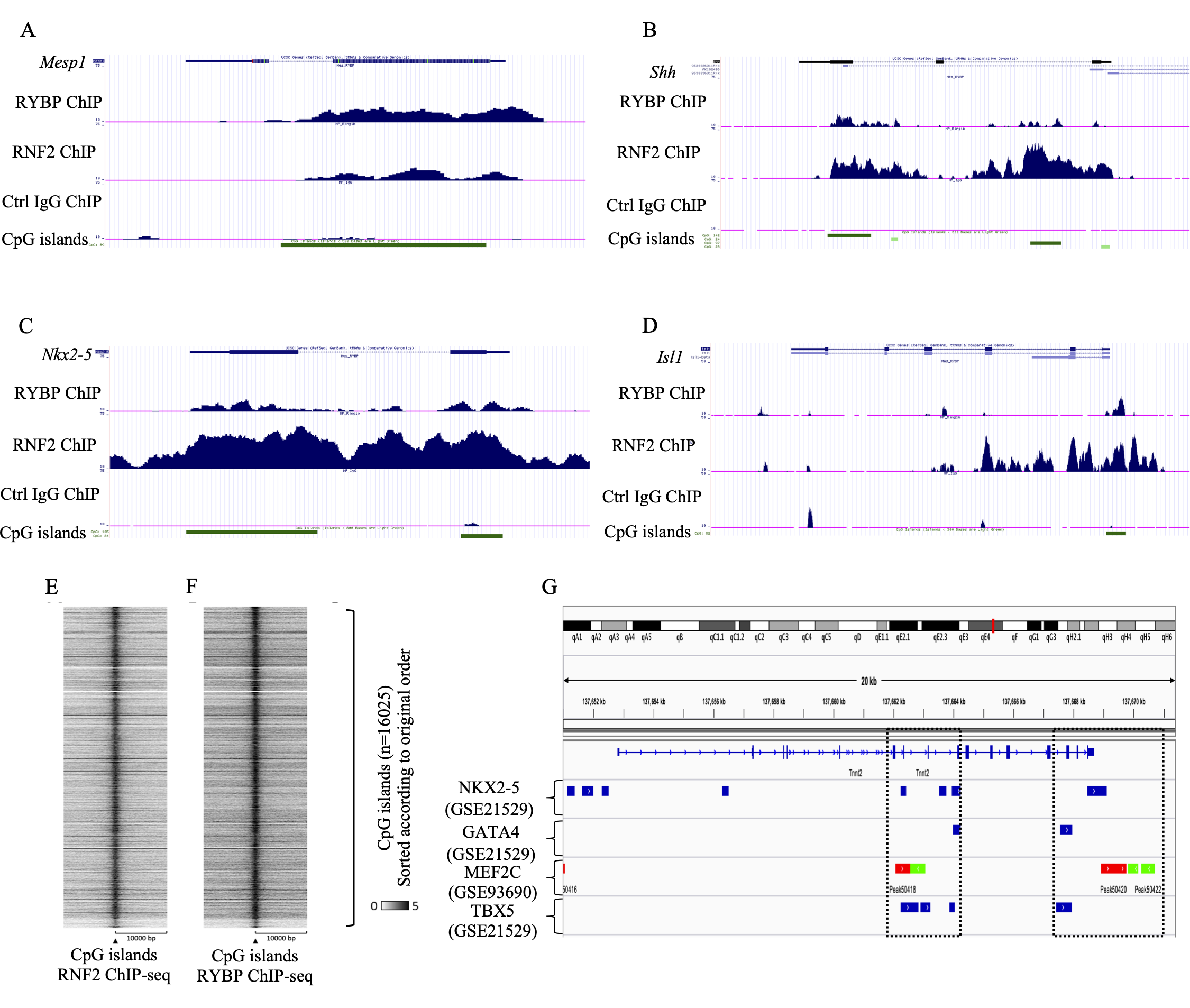

Supplement: S2 Fig — (TIF) [file pone.0235922.s002.tif]
